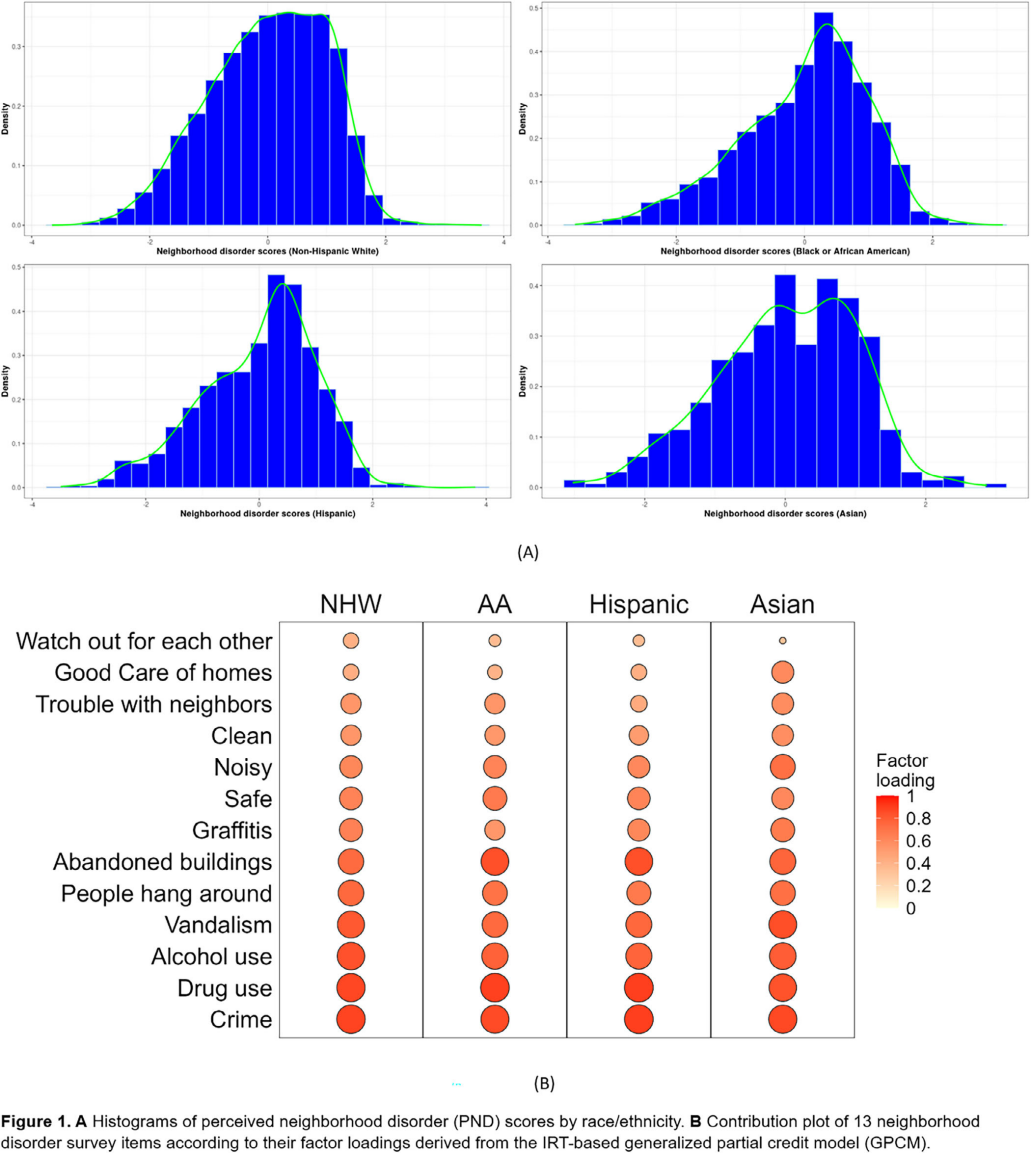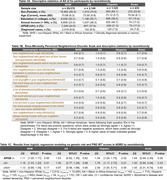# Joint Association of Genetic Risk and Perceived Neighborhood Disorder with Dementia Across Diverse Populations: Results from All of Us

**DOI:** 10.1002/alz70855_107428

**Published:** 2025-12-25

**Authors:** Xian Wu, Jing Zhang, Inori Tsuchiya, Yucong Sang, Jordan Brown, Khine Zin Aung, Yuriko Katsumata, Erin L. Abner, Peter T Nelson, Bryan D James, David W. Fardo

**Affiliations:** ^1^ Department of Biostatistics, College of Public Health, University of Kentucky, Lexington, KY, USA; ^2^ Sanders‐Brown Center on Aging, University of Kentucky, Lexington, KY, USA; ^3^ College of Nursing, University of Kentucky, Lexington, KY, USA; ^4^ EVPHA Information Technology, University of Kentucky, Lexington, KY, USA; ^5^ Department of Sociology, University of Kentucky, Lexington, KY, USA, Lexington, KY, USA; ^6^ Department of Epidemiology and Environmental Health, University of Kentucky, Lexington, KY, USA; ^7^ Department of Pathology, University of Kentucky, Lexington, KY, USA; ^8^ Department of Internal Medicine Rush University Medical Center, Chicago, IL, USA; ^9^ Rush Alzheimer's Disease Center, Chicago, IL, USA

## Abstract

**Background:**

Both genetic and social factors contribute to Alzheimer's disease and related dementia (ADRD) risk. Genetic risk may be modified by social factors, such as neighborhood characteristics. While previous studies have commonly constructed social factor scores by summing item scores, item response theory (IRT) can estimate more precise composite scores by modeling observable items. This study aimed to 1) construct perceived neighborhood disorder (PND) scores using IRT models across diverse populations, and 2) examine their interaction with genetic risk in ADRD.

**Method:**

Using All of Us data, we constructed four cohorts (aged 65+ years): non‐Hispanic White (NHW), Black or African American (AA), Hispanic, and Asian. We applied an IRT‐based generalized partial credit model to constructed PND scores within each cohort, using the 13‐item Ross‐Mirowsky Perceived Neighborhood Disorder Scale. Higher scores indicated greater neighborhood disadvantage. *APOE* ε4 status as a measure of genetic risk for ADRD. Clinically diagnosed dementia or memory impairment was the phenotype: case (diagnosed) and control (non‐diagnosed). Logistic regression was employed to estimate joint associations among PND scores and *APOE* ε4 status with dementia, adjusting for age and sex. In this analysis, we applied random sampling to balance sample sizes between cases and controls.

**Result:**

7∼8% of participants reported clinically diagnosed dementia or memory impairment (Table 1A). *APOE* ε4 burden was highest among AA (38%), followed by NHW (25%), Hispanic (21%), and Asian (17%). PND item scores varied across groups. For instance, AA and Hispanic participants perceived greater neighborhood safety disadvantages compared to NHW and Asian cohorts (Table 1B). PND scores were approximately normally distributed (Figure 1A). Crime, drug use, alcohol use, and vandalism were the most informative items (Figure 1B). Among NHW participants without ε4 or with one copy of ε4, a 1‐point PND score increase raised dementia odds by 1.11 (‐/‐) and 1.18 (ε4/‐). In Hispanics, as 1‐point PND score increased, odds of dementia increased by 1.34 (‐/‐), 1.78 (ε4/‐), and 2.39 (ε4/ε4), respectively (Table 1C).

**Conclusion:**

*APOE* and PND were jointly associated with dementia risk. Future research should include additional social measures, more genes, ADRD subtypes, and larger sample sizes to understand gene‐environment interaction in dementia.